# Inferior performance of established and novel serum inflammatory markers in diagnosing periprosthetic joint infections

**DOI:** 10.1007/s00264-020-04889-z

**Published:** 2020-11-27

**Authors:** Irene Katharina Sigmund, Johannes Holinka, Kevin Staats, Florian Sevelda, Richard Lass, Bernd Kubista, Alexander Giurea, Reinhard Windhager

**Affiliations:** Department of Orthopedics and Trauma Surgery, Medical University of Vienna, Spitalgasse 23, 1090 Vienna, Austria

**Keywords:** Periprosthetic joint infection, Diagnosis, Serum inflammatory markers, CRP, Fibrinogen, Platelet count to mean platelet volume ratio

## Abstract

**Purpose:**

Besides other diagnostic test methods, established serum inflammatory markers such as serum C-reactive protein or leukocyte count are widely used preoperatively to aid in diagnosing periprosthetic joint infections (PJI). Although low accuracies were reported, these parameters are easily accessible and routinely available. Novel biomarkers with promising results in diagnosing PJI (platelet count to mean platelet volume ratio) or other infectious conditions (percentage of neutrophils, neutrophils to lymphocytes ratio) were described. The purpose of this study was to investigate the diagnostic value of established and novel serum inflammatory biomarkers for the diagnosis of PJI so as to compare the results to find the serum inflammatory marker with the best performance.

**Methods:**

In 177 patients with a previous total hip (*n* = 91) or knee (*n* = 86) arthroplasty and indicated revision surgery, the diagnostic value of the routinely available serum inflammatory markers C-reactive protein (CRP), white blood cell count (WBC), percentage of neutrophils (%N), neutrophils to lymphocytes ratio (NLR), fibrinogen and platelet count to mean platelet volume ratio (PC/mPV) were examined retrospectively via receiver operating characteristic curve analysis (AUC). The curves were compared using the *z*-test.

**Results:**

Sensitivities of serum CRP, WBC, %N, NLR, fibrinogen and PC/mPV were calculated with 68%, 36%, 66%, 63%, 69% and 43%, respectively. Specificities were 87%, 89%, 67%, 73%, 89% and 81%, respectively. Serum CRP (0.78) and fibrinogen (0.79) showed significantly better AUCs compared with serum WBC (0.63), %N (0.67), NLR (0.68) and PC/mPV (0.62) (*p* < 0.0001). Patients with PJI caused by a low-virulent microorganism (median CRP: 17.6 mg/L) obtained lower CRP levels compared with infections caused by high-virulent microorganisms (median CRP: 49.2 mg/L; *p* = 0.044). The combination of CRP and fibrinogen showed a better sensitivity (77%) with similar specificity (83%) than one method alone but not at a significant level (CRP (*p* = 0.200); fibrinogen (*p* = 0.437)).

**Conclusion:**

Serum CRP and fibrinogen showed the best accuracies among these widely available serum inflammatory parameters. However, due to the insufficient performance, these biomarkers can only be recommended as suggestive criteria in diagnosing PJI. The preoperative workup should always be complemented by more specific tests such as synovial fluid analysis.

## Introduction

Elevated serum inflammatory biomarkers, such as serum C-reactive protein (CRP) and white blood cell count (WBC), can be the first indication or occasionally the only pre-operative indication of periprosthetic joint infections (PJI) when other clinical symptoms (such as joint effusion, pain, swelling, redness) are missing.

However, due to the low specificity, these tests cannot be utilized alone as confirmatory criteria. They need to be complemented by more specific tests such as synovial fluid analysis (leukocyte count, percentage of polymorphonuclear neutrophils), microbiology (including sonication) and histology of deep tissue samples. These latter diagnostic methods are, however, time-consuming, resource intensive and invasive. The results of microbiology and histology of deep tissue samples are only available post-operatively. Therefore, there is no pre-operative diagnostic value of these tests.

Serum inflammatory markers provide pre-operative information, deliver timely results, are cheap, easy to use, and are widely available around the world. Nonetheless, for a sufficient pre-operative diagnosis novel, more accurate serum parameters are needed. In a recently published study, attention has focused on the platelet count to mean platelet volume ratio (PC/mPV) with an accuracy of 75% [1]. The combination of ESR, CRP and PC/mPV yielded an even higher accuracy of 82%. Therefore, it could be a promising adjunct in diagnosing PJI, pre-operatively.

Other potential serum inflammatory markers which have been correlated with infection in other infectious conditions are fibrinogen [2, 3], the percentage of neutrophils (%N) and the neutrophils to lymphocytes ratio (NLR) [4–7]. While the performance of %N and NLR was not investigated previously, serum fibrinogen was shown to be highly sensitive (90%) but unspecific (34%) for detecting PJI [8].

The aim of our study was to evaluate the accuracy of the easily accessible and routinely available established serum inflammatory biomarkers C-reactive protein, leukocyte count and fibrinogen and the novel and also routinely available serum biomarkers platelet count to mean platelet volume ratio, percentage of neutrophils and neutrophils to lymphocytes ratio in the diagnosis of periprosthetic joint infections when using the European Bone and Joint Infection Society (EBJIS) criteria (McNally MA, Sousa R, Wouthuyzen-Bakker M, Chen AF, Soriano A, Vogely CH, Clauss M, Higuera-Rueda CA, Trebse R. The EBJIS Definition of Prosthetic Joint Infection: a practical guide for clinicians. Bone Joint J 2020; in press). In addition, a comparison among these parameters was done to find the serum inflammatory marker with the best performance.

## Materials and methods

### Study design

This retrospective study was conducted at a single tertiary healthcare centre in accordance with the Declaration of Helsinki after local institutional ethical review board approval (EK1455/2019). Patients with an indicated revision surgery after a total hip arthroplasty or total knee arthroplasty were included from January 2015 to June 2019. Patients without pre-operative or sufficient blood analysis were excluded, as well as patients with surgery within the last six weeks, a joint aspiration with a cement spacer in place, a resection arthroplasty or a second stage of two stage revision. Demographics and results of serum inflammatory markers, synovial fluid analysis, histology and microbiology were recorded.

The European Bone and Joint Infection Society (EBJIS) criteria were used to define a periprosthetic joint infection (McNally MA, Sousa R, Wouthuyzen-Bakker M, Chen AF, Soriano A, Vogely CH, Clauss M, Higuera-Rueda CA, Trebse R. The EBJIS Definition of Prosthetic Joint Infection: a practical guide for clinicians. Bone Joint J 2020; in press).

### Determination of diagnostic tests

At our institution, a standardized workup is performed to aid in diagnosing periprosthetic joint infection. Of all patients, blood samples were taken, pre-operatively. For serum CRP analysis, plasma was stored in lithium-heparin vacuum collection tubes. Automated laboratory spectrophotometric analysis was done to quantify CRP (Cobas®8000, Roche Diagnostics GmbH, Vienna, Austria). The established laboratory cutoff of ≥ 10 mg/L was used [9]. The results of serum WBC, differential, platelet count and mean platelet volume were measured in the pre-operative complete blood count. For quantification of WBC and differential (to calculate %N and NLR), ethylenediaminetetraacetic acid (EDTA) plasma was analysed by automated flow cytometry (Sysmex XN® analyser, Sysmex Austria GmbH, Vienna, Austria). For association with infection, the established cutoff of ≥ 10 × 10^9 white blood cells/L was seen as positive. Plate count and median plate volume are evaluated using impedance measurements via Sysmex XN® and Sysmex XE® analyser as previously described [1]. Pre-operatively, serum fibrinogen is also routinely analysed for coagulation analysis using coagulometry with sodium citrate blood (STA R Max2®, Diagnostica Stago, Asnières sur Seine, France).

In addition, synovial fluid of the affected joint was aspirated under sterile conditions and analysed for leukocyte count, granulocyte percentage and qualitative alpha defensin and sent for microbiological investigations as previously described [10]. For histopathological analysis, at least two periprosthetic tissue specimens were collected, processed and classified according to the Krenn criteria by default [11]. For microbiological analysis, at least three tissue specimens were processed per standard laboratory protocol with cultures held for 14 days [12, 13]. The explanted components of the prosthesis were sent for sonication culture analysis.

For detailed analysis, the patients were stratified into two groups according to the virulence of the causative microorganism: the high-virulence group (infection caused by, e.g. *Staphylococcus aureus*, enterococci, *Enterobacteriaceae*, streptococci) and the low-virulence group (e.g. coagulase-negative staphylococci, *Cutibacterium* spp.). If a polymicrobial infection caused by high- and low-virulence pathogens was present, the patient was assigned to the high-virulence group.

### Statistical analysis

The performance of each serum inflammatory marker was assessed by calculating sensitivity, specificity, accuracy, positive (PPV) and negative (NPV) predictive value, positive (LR+) and negative likelihood ratio (LR−), area under the ROC curve (AUC) and their 95% confidence intervals. The laboratory cutoffs of %N, NLR, PC/mPV and fibrinogen were assessed via receiver operating characteristic (ROC) curve analysis. For comparison analysis, the calculated AUCs of each test were compared using the *z*-test. For performance analysis, the test method of interest was excluded from the infection definition to avoid incorporation bias. Statistical analyses were performed in XLSTAT statistical and data analysis solution (Version 2019.3.2.; Addinsoft 2020, Boston, USA).

## Results

### Demographics

A total of 177 patients with an indicated revision surgery after a total joint arthroplasty were included. The median age of the whole study cohort was 73 years (range: 22–93 years) and 108 patients (61%) were female. Revision surgery was performed on 91 total hip (51%) and on 86 total knee replacements (49%). Eight of the nine patients treated with antibiotics pre-operatively were identified as infected (*p* = 0.002).

On the basis of the EBJIS criteria, 75 cases (42%) were defined as septic and 102 (58%) as aseptic. Regarding age, gender and localization, no difference between both groups was observed. There were significantly higher levels of serum inflammatory markers in the septic group (*p* ≤ 0.001; Table 1). The sensitivity, specificity and AUC of microbiology were 60.8% (95% CI: 49.4–71.1), 98.0% (92.6–99.9) and 0.794 (0.737–0.852); of histology 92.0% (83.2–96.5), 100% (95.5–100) and 0.960 (0.929–0.991) and of synovial fluid white blood cell count 78.8% (65.7–87.8), 97.4% (85.4–100) and 0.881 (0.820–0.943), respectively. Histology and synovial fluid leucocyte count were significantly better than serum CRP, serum WBC, %N, NLR, serum fibrinogen and PC/mPV (*p* < 0.0001).

**Table 1:** Demographics of all included patients

Demographics	Septic cases (*n* = 75)	Aseptic cases (*n* = 102)	*p* value	Total (*n* = 177)
Age (range)	72 (22–93)	74 (28–90)	0.246°	73 (22–93)
Female gender (%)	43 (57)	65 (64)	0.389*	108 (61)
Localization (%)				
Hip	36 (48)	55 (54)	0.436*	91 (51)
Knee	39 (52)	47 (46)	0.436*	86 (49)
Antibiotics (%)	8 (11)	1 (1)	0.002*	9 (5)
Serum inflammatory markers				
CRP (range) (*n* = 176)	28.4 (0.8–539.6)	3.2 (0.3–47.6)	< 0.0001°	5.4 (0.3–539.6)
WBC (range) (*n* = 176)	8.8 (4.1–21.0)	7.0 (3.5–15.6)	< 0.0001°	7.3 (3.5–21.0)
%N (range) (*n* = 162)	72.1 (54.1–93.9)	65.4 (41.0–89.5)	< 0.0001°	68.5 (41.0–93.9)
NLR (range) (*n* = 162)	4.0 (1.0–44.7)	3.0 (0.9–18.1)	0.001°	3.4 (0.9–44.7)
Fibrinogen (range) (*n* = 169)	540 (207–1152)	362 (148–681)	< 0.0001°	388 (148–1152)
PC/mPV (range) (*n* = 176)	25.1 (10.7–97.5)	23.1 (9.5–54.9)	0.001°	23.7 (9.5–97.5)

*CRP*, serum C-reactive protein (mg/L); *WBC*, serum white blood cell count (cells/L); *%N*, percentage of neutrophils (%); *NLR*, neutrophils to lymphocytes ratio, fibrinogen (mg/dL); *PC/mPV*, platelet count to mean platelet volume ratio (PC/mPV)

°Student’s *t* test

*Chi-squared test

### Accuracy of serum inflammatory biomarkers

The performances of all evaluated serum inflammatory parameters are illustrated in Table 2. According to the ROC curve analysis, the optimal %N cutoff was ≥ 69.3% with a sensitivity of 65.7% (53.7–75.9) and specificity of 67.4% (57.4–76.0). The optimal NLR cutoff was ≥ 3.82 with a sensitivity of 62.7% (50.7–73.3) and specificity of 72.6% (62.8–80.6). The optimal threshold of serum fibrinogen was ≥ 457 mg/dL showing a sensitivity of 68.5% (57.1–78.0) and specificity of 88.5% (80.4–93.6). The optimal PC/mPV ratio was ≥ 29.4 with a sensitivity of 42.7% (32.1–54.0) and specificity of 81.2% (72.3–87.7), respectively.

**Table 2:** Sensitivity, specificity, accuracy, positive (PPV) and negative (NPV) predictive value, positive (LR+) and negative (LR−) likelihood ratio and area under the curve (AUC) of serum C-reactive protein (CRP), serum white blood cell count (WBC), percentage of neutrophils (%N), neutrophils to lymphocytes ratio (NLR), fibrinogen and platelet count to mean platelet volume ratio (PC/mPV)

	CRP (*n* = 176)	WBC (*n* = 176)	%N (*n* = 162)	NLR (*n* = 162)	Fibrinogen (*n* = 169)	PC/mPV (*n* = 176)
Cutoff	≥ 10 mg/L	≥ 10 × 10^9 cells/L	≥ 69.3%	≥ 3.82	≥ 457 mg/dL	≥ 29.4
Sensitivity (%)	68.0 (56.7–77.5)	36.0 (26.1–47.3)	65.7 (53.7–75.9)	62.7 (50.7–73.3)	68.5 (57.1–78.0)	42.7 (32.1–54.0)
Specificity (%)	87.1 (79.0–92.4)	89.1 (81.3–93.9)	67.4 (57.4–76.0)	72.6 (62.8–80.6)	88.5 (80.4–93.6)	81.2 (72.3–87.7)
Accuracy (%)	79.0 (73.0–85.0)	66.5 (59.5–73.5)	66.7 (59.4–73.9)	68.5 (61.4–75.7)	79.9 (73.8–85.9)	64.8 (57.7–71.8)
PPV (%)	79.7 (69.8–89.5)	71.1 (56.6–85.5)	58.7 (47.5–69.8)	61.8 (50.2–73.3)	82.0 (72.3–91.6)	62.7 (49.5–76.0)
NPV (%)	78.6 (71.0–86.2)	65.2 (57.3–73.2)	73.6 (64.3–82.8)	73.4 (64.5–82.3)	78.7 (71.0–86.4)	65.6 (57.3–73.9)
LR+	5.283 (3.108–8.981)	3.305 (1.753–6.233)	2.013 (1.437–2.819)	2.290 (1.573–3.336)	5.978 (3.355–19.649)	2.268 (1.400–3.675)
LR−	0.367 (0.262–0.515)	0.718 (0.598–0.862)	0.510 (0.356–0.730)	0.514 (0.368–0.717)	0.356 (0.252–0.503)	0.706 (0.569–0.877)
AUC	0.776 (0.713–0.838)	0.626 (0.563–0.688)	0.665 (0.591–0.740)	0.677 (0.603–0.750)	0.785 (0.723–0.848)	0.619 (0.551–0.687)

**Fig. 1:**
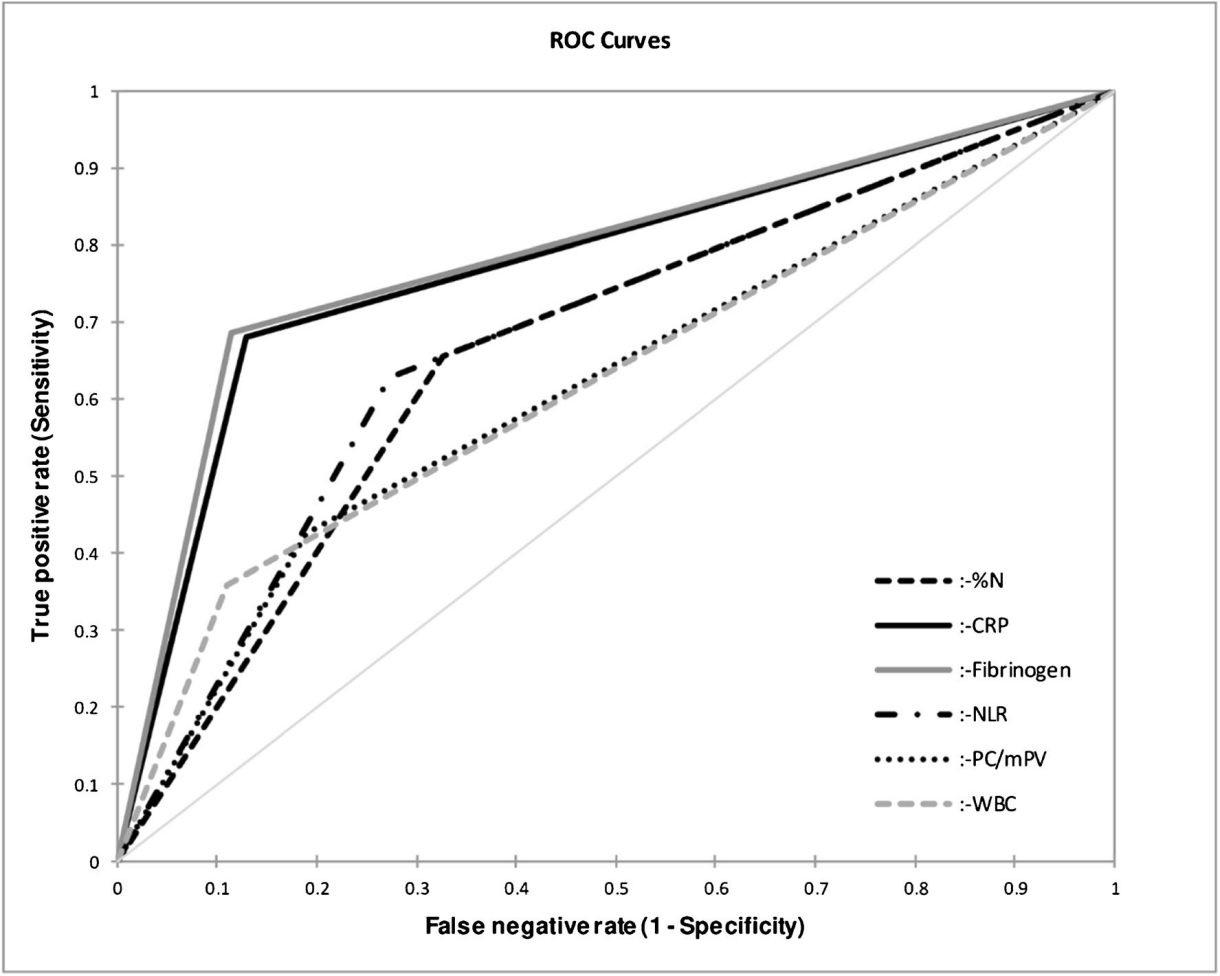
Receiver operating characteristic curves for accuracy of periprosthetic joint infection based on the percentage of neutrophils (%N), serum C-reactive protein (CRP), fibrinogen, neutrophils to lymphocytes ratio (NLR), platelet count to mean platelet volume ratio (PC/mPV) and white blood cell count (WBC)

**Fig. 2:**
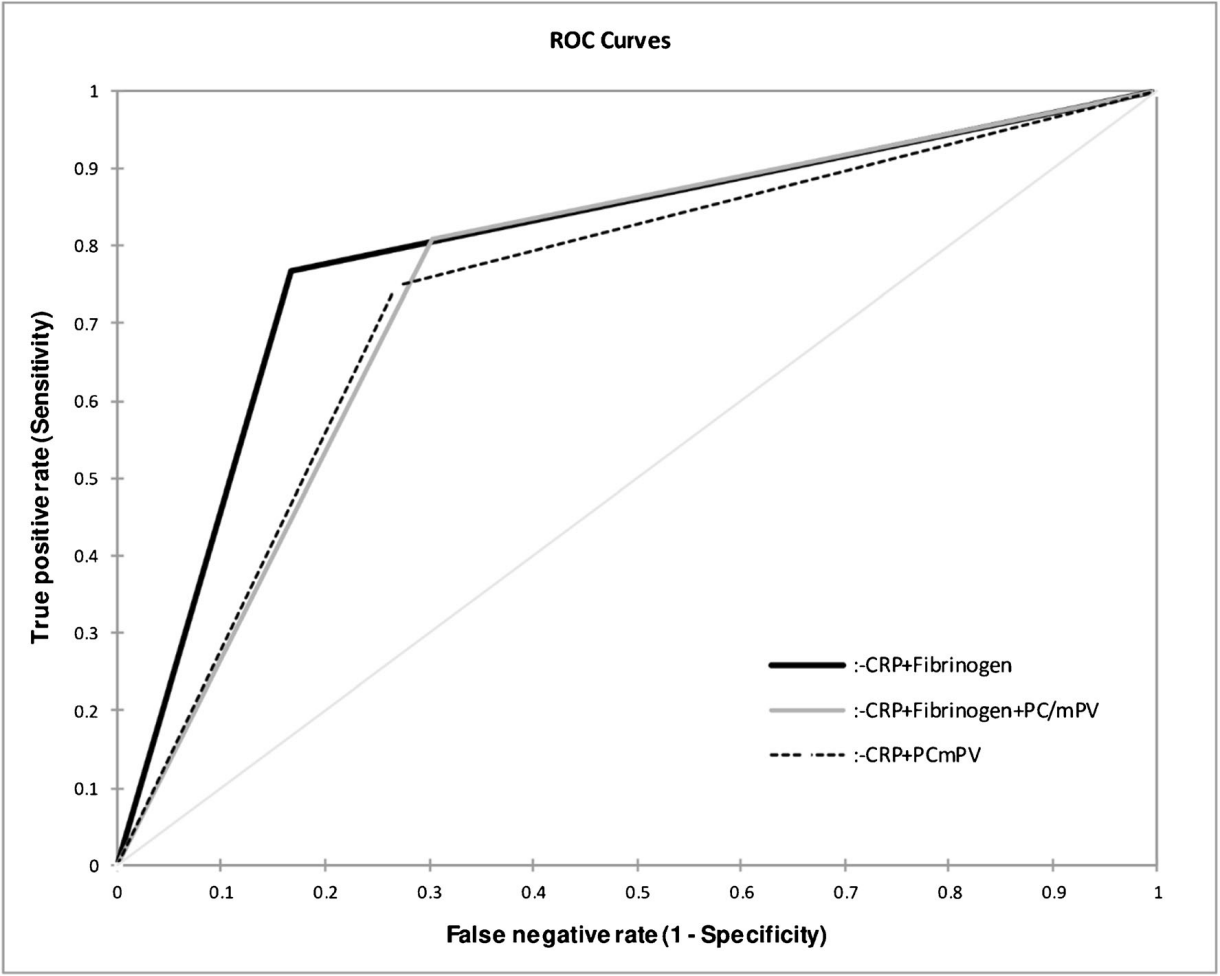
Receiver operating characteristic curves for accuracy of periprosthetic joint infection based on serum C-reactive protein (CRP) combined with fibrinogen, CRP combined with fibrinogen and platelet count to mean platelet volume ratio (PC/mPV) and CRP combined with PC/mPV

**Fig. 3:**
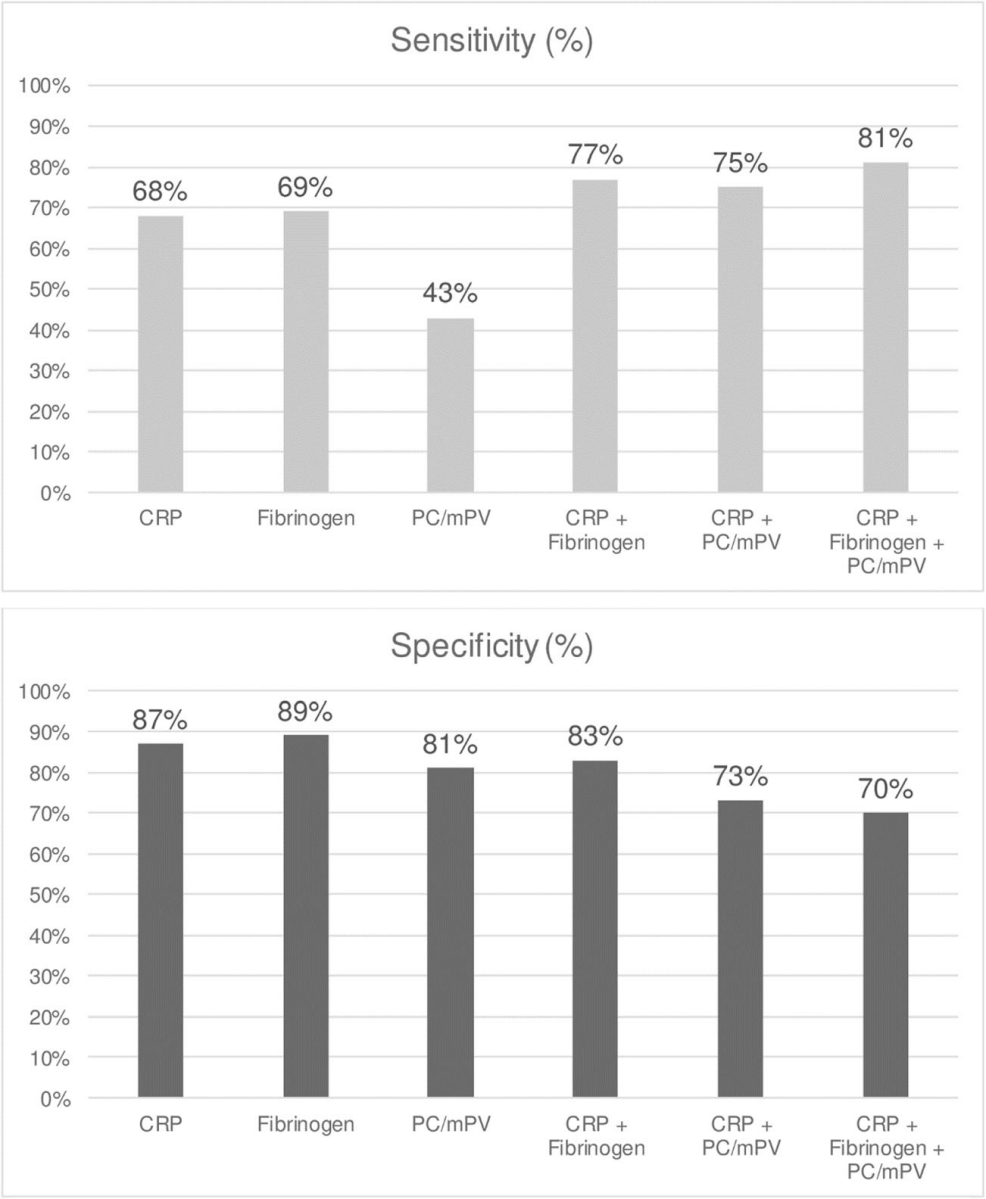
Comparison of sensitivities and specificities between serum C-reactive protein (CRP), fibrinogen, platelet count to mean platelet volume ratio (PC/mPV) and their combinations. Serum CRP combined with fibrinogen shows the best accuracy (combination of sensitivity and specificity) among these test methods

According to the comparison analysis (Fig. 1), serum CRP and fibrinogen showed a significantly better performance than WBC (*p* < 0.0001), %N (*p* < 0.0001), NLR (*p* < 0.0001) and PC/mPV (*p* < 0.0001). No difference was observed between serum CRP and fibrinogen (*p* = 0.620). If these latter two biomarkers were combined (Fig. 2), sensitivity increased to 76.7% (65.7–85.0) but specificity decreased to 83.3% (74.5–89.5) (Table 3). This combination showed a better accuracy than WBC (*p* < 0.0001), NLR (*p* < 0.0001), %N (*p* < 0.0001), PC/mPV (*p* < 0.0001), the combination of CRP with fibrinogen and PC/mPV (*p* = 0.016) and CRP combined with PC/mPV (*p* = 0.002) (Fig. 3). However, this combination was not better than one method alone (CRP (*p* = 0.200), fibrinogen (*p* = 0.437)).

**Table 3:** Sensitivity, specificity, accuracy, positive (PPV) and negative (NPV) predictive value, positive (LR+) and negative (LR−) likelihood ratio and area under the curve (AUC) of serum C-reactive protein (CRP) combined with fibrinogen, CRP combined with platelet count to mean platelet volume ratio (PC/mPV) and CRP combined with fibrinogen and PC/mPV

	CRP + fibrinogen	CRP + PC/mPV	CRP + fibrinogen + PC/mPV
Sensitivity (%)	76.7 (65.7–85.0)	74.7 (63.7–83.2)	80.8 (70.2–88.3)
Specificity (%)	83.3 (74.5–89.5)	73.3 (63.8–80.9)	69.8 (59.9–78.1)
Accuracy (%)	80.5 (74.5–89.5)	73.9 (67.4–80.4)	74.6 (68.0–81.1)
PPV (%)	77.8 (68.2–87.4)	67.5 (57.4–77.5)	67.0 (57.2–76.9)
NPV (%)	82.5 (74.9–90.0)	79.6 (71.4–87.8)	82.7 (74.5–91.0)
LR+	4.603 (2.892–7.326)	2.793 (1.971–3.959)	2.675 (1.935–3.699)
LR−	0.279 (0.183–0.428)	0.346 (0.230–0.519)	0.275 (0.169–0.448)
AUC	0.800 (0.739–0.862)	0.740 (0.674–0.806)	0.753 (0.688–0.818)

### Microorganisms

In the septic group, microbial growth was observed in 46 (46/75, 61%) patients including 44 (96%) monomicrobial and two (4%) polymicrobial infections. The most common microorganism was *Staphylococcus aureus* (*n* = 12), followed by coagulase-negative staphylococci (*n* = 11), streptococci (*n* = 7) and *Enterobacteriaceae* (*n* = 5). In 29 (29/75, 39%) patients with PJI, no microorganism was detected.

**Fig. 4:**
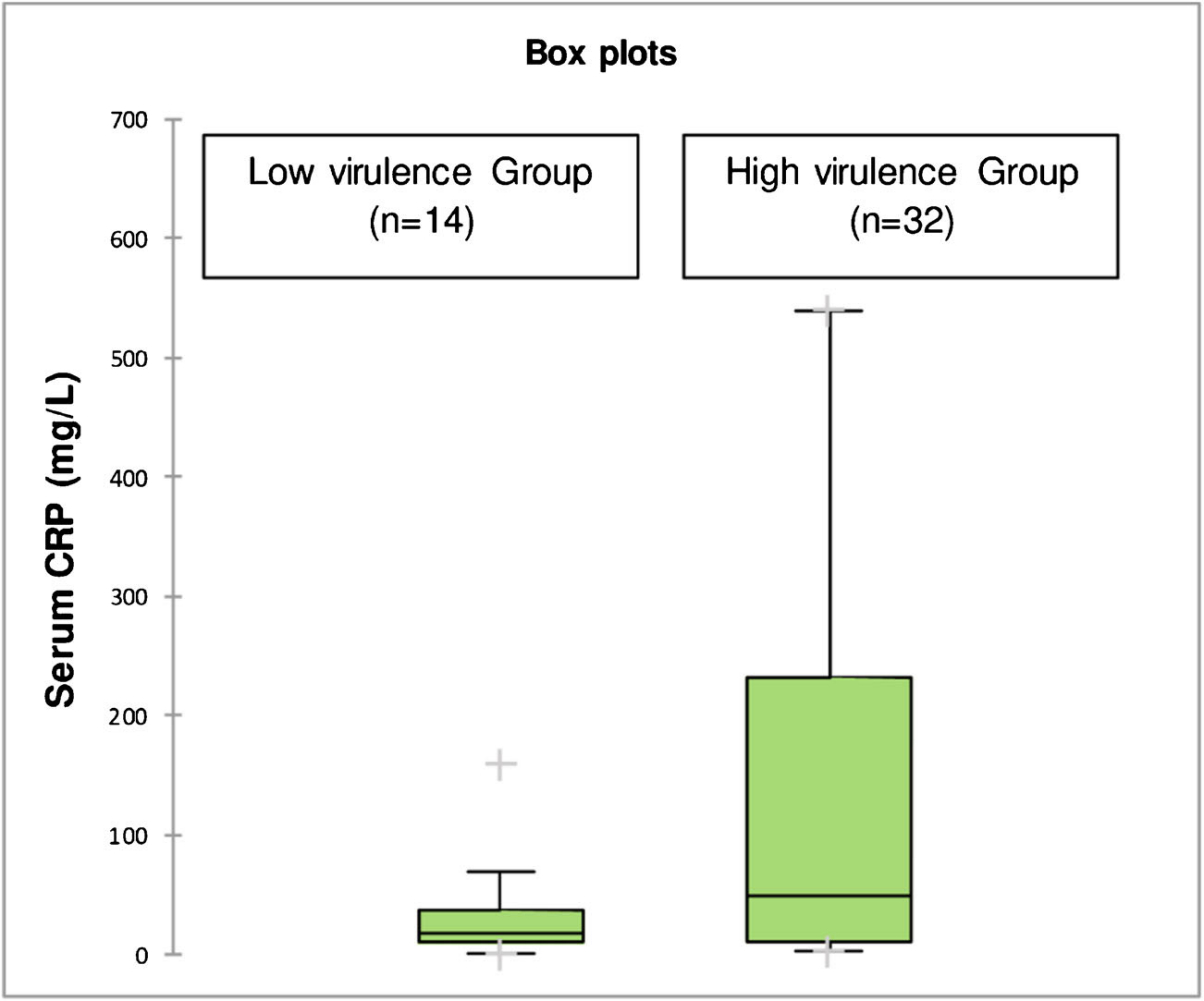
Boxplots of serum C-reactive protein (CRP) levels depending on the virulence of the causing microorganism. The horizontal line represents the median CRP level, the black box the interquartile range, the whiskers the minimum and maximum and the cross outliners

Fourteen (14/46; 30%) of the 46 patients with a culture-positive PJI were stratified into the low-virulence group and the remainder 32 (32/36; 70%) patients into the high-virulence group. The median serum CRP level was 17.6 mg/L (IQR 9.5–36.9) in the low-virulence group and 49.2 mg/L (IQR 10.9–231.9) in the high-virulence group (Fig. 4). A statistically significant difference was shown between these groups with lower CRP levels in patients with an infection caused by a low-virulence organism (*p* = 0.044).

**Fig. 5:**
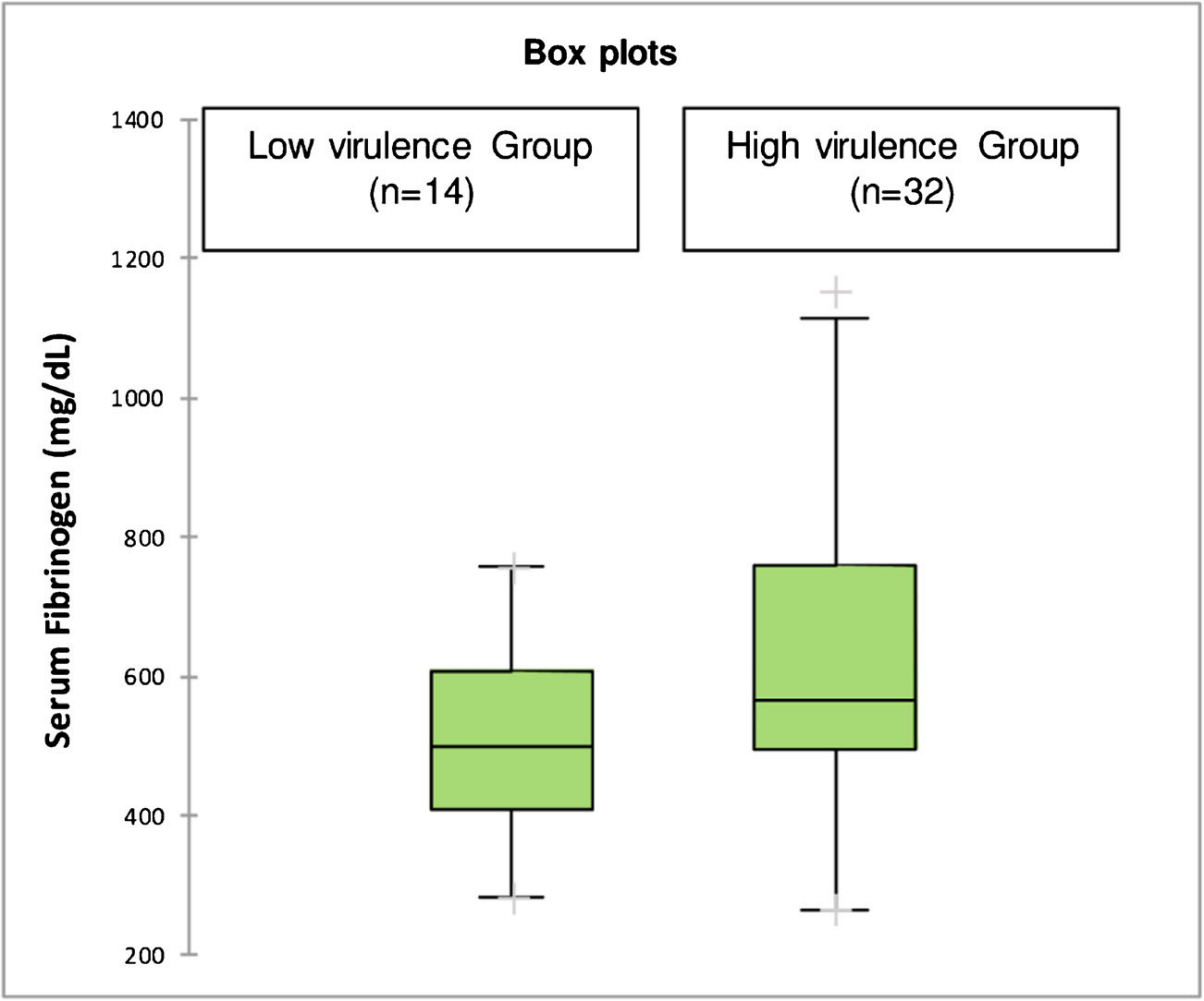
Boxplots of serum fibrinogen levels depending on the virulence of the causing microorganism. The horizontal line represents the median CRP level, the black box the interquartile range, the whiskers the minimum and maximum and the cross outliners

The median serum fibrinogen level was 499 mg/dL (IQR 409–609) in the low-virulence group and 567 mg/dL (IQR 496–758) in the high-virulence group (Fig. 5). Regarding serum fibrinogen, no difference was calculated between both groups (*p* = 0.111).

## Discussion

Overall, this study demonstrates an insufficient performance of established and novel serum inflammatory biomarkers in diagnosing a periprosthetic joint infection when applying the EBJIS criteria.

Although serum CRP showed the best accuracy among the evaluated serum parameters, sensitivity (68%) and specificity (87%) were only moderate. These results are similar to the reported data in the literature with sensitivities ranging from 74 to 90% and specificities from 71 to 88% [1, 8, 14, 15], respectively. The low sensitivity could be explained by the low serum CRP concentration in patients with an infection caused by a low-virulence microorganism capable of forming biofilm. This biofilm protects the pathogen against the host immune system resulting in a weakened immune response and, hence, reduced release of inflammatory biomarkers [16]. An infection could be misdiagnosed due to a lack of systemic inflammation. Although the number of infections with a detected microorganism was small in our study (*n* = 46), we observed a significantly lower serum CRP level in the low-virulence group (*p* = 0.044), which is in line with the currently available literature. In a study by Perez-Prieto et al. [17], 23 of 73 (32%) patients with a culture-positive PJI had a normal serum CRP level pre-operatively. Of these 23 patients, 16 (16/23; 70%) infections were caused by a low-virulence microorganism. In addition, Akgün et al. showed similar results [18]. Of 215 culture-positive PJIs, 77 (36%) had a normal serum CRP concentration, and 66 (66/77; 86%) were caused by low-virulence organisms. These findings highlight the high false-negative rate when using serum CRP in the diagnosis of PJI.

Serum CRP also showed false-positive cases and, hence, a reduced specificity in our study, resulting in a potential overtreatment with unnecessary surgical revisions and prolonged antimicrobial treatment if used alone. An explanation could be the fact that CRP is a systemic parameter influenced by other systemic conditions or inflammations (autoimmune disorders (e.g. rheumatoid arthritis), other infectious foci (e.g. bronchitis, pneumonia, urinary tract infections) or cancer) misleading the diagnosis of PJI. Overall, our results underline the insufficient accuracy of serum CRP in diagnosing PJI.

Serum fibrinogen is well known for its role in the coagulation cascade and has also been correlated with infection in other conditions such as appendicitis [2] and sepsis [3]. It showed an impact on the inflammation process by inducing and promoting the synthesis of proinflammatory cytokines (interleukin-6 and tumour necrosis factor α) in mononuclear cells [19] and activating various immune cells [20]. Klim et al. [8] analysed serum fibrinogen in 84 patients with a suspected PJI and showed a high sensitivity of 90%, but very poor specificity of only 34% when a cutoff of 519 mg/dL was applied. In the study by Alturfan et al. [21], a good sensitivity of 93% and specificity of 86% was reported when using a cutoff of 432 mg/dL. However, in our study, serum fibrinogen showed a lower sensitivity (69%), but similar specificity (89%) at an optimal cutoff level of ≥ 457 mg/dL determined by ROC curve analysis. It was comparable to serum CRP (*p* = 0.620) and significantly better than WBC, %N, NLR and PC/mPV (*p* < 0.0001). However, the overall accuracy of this method alone is also insufficient to confirm or exclude infection and should, therefore, be only used as a suggestive criterion. Additionally, the combination of serum CRP and fibrinogen showed an improved sensitivity (77%) and nearly similar specificity (83%) than one method alone but not at a statistically significant level (*p* = 0.200). Nevertheless, the accuracy was only moderate; hence, this combination cannot be used for PJI confirmation.

Another easily accessible and routinely available parameter is the ratio of platelet count to mean platelet volume (PC/mPV). It has been shown that the platelet count (PC) increases and the median platelet volume (mPV) decreases during episodes of bacterial infections [22, 23] resulting in an increased ratio. Paziuk et al. [1] evaluated PC/mPV in a cohort of 5888 patients with revision total hip and knee arthroplasties including 949 (16%) septic cases. They reported a sensitivity of 48% and specificity of 81% when using a cutoff of 31.7. However, PC/mPV showed the highest specificity compared with serum CRP (74%) and serum erythrocyte sedimentation rate (ESR, 78%) in their study. In addition, the combination of these three parameters showed a significantly improved accuracy (*p* < 0.05). However, sensitivity (80%) and specificity (82%) were—in our opinion—still insufficient to confirm or exclude an infection. In our study, sensitivity (43%) and specificity (81%) of PC/mPV alone showed inferior results in comparison with CRP and fibrinogen (*p* < 0.0001) when using the optimal cutoff of ≥ 29.4. Hence, PC/mPV cannot be considered as sufficient test method.

Due to the low accuracy of ESR in previous studies [24], it is not routinely determined in our institution. Hence, we were not able to analyse the combined performance of PC/mPV, CRP and ESR as described by Paziuk et al. [1]. Instead, we evaluated the combination of PC/mPV, CRP and fibrinogen. While sensitivity (81%) improved, specificity (70%) decreased (Fig. 3). However, serum inflammatory biomarkers are typically known as sensitive and are—as systemic parameters—usually unspecific. Since our aforementioned combination showed a lower specificity, we cannot recommend it as an additional tool in diagnosing PJI.

Finally, serum WBC is known for its good specificity (87–94%) but poor sensitivity (21–45%) [25–27]. Applying EBJIS criteria, we could confirm this inferior performance of WBC (sensitivity 36%, specificity 89%) in our study. Percentage of neutrophils and the neutrophils to lymphocytes ratio showed promising results in diagnosing other infectious conditions such as appendicitis, surgical site infections or bloodstream infections [4, 5, 7] but could only obtain moderate results when diagnosing PJI. With sensitivities of about 60% and specificities of about 70%, these parameters did not aid in diagnosing PJI.

Our evaluated serum inflammatory markers provide preoperative information, deliver timely results, are cheap, easy to use, are widely available and can initiate further diagnostic analysis. To the best of our knowledge, a comparison between these analysed serum inflammatory biomarkers was not done previously when using the EBJIS criteria. The novel markers %N, NLR and PC/mPV showed an inferior diagnostic value in comparison with the established markers: CRP and fibrinogen. Therefore, CRP and fibrinogen should remain the main serum inflammatory biomarkers in routine clinical practice to aid in diagnosing PJI. However, they can only be used as suggestive criteria in the preoperative diagnosis of PJI due to their insufficient accuracy. However, a standardized pre-operative workup should always be complemented by more specific tests such as synovial fluid analysis. In this study, synovial fluid leukocyte count showed a good accuracy and a significantly better performance than all these serum inflammatory parameters (*p* ≤ 0.0001) and can, therefore, be recommended as a confirmatory criterion in the diagnosis of PJI.

This study is limited by its retrospective design and associated disadvantages. Some parameters listed in the EBJIS criteria were not always available for all cases (Table 1) which is known to be reality in clinical routine [28]. In this study, we used the EBJIS criteria which can detect more periprosthetic joint infections, compared with other infection definition criteria [29]. However, this definition can be prone to misdiagnose an aseptic case as infection. On the other hand, the commonly used Musculoskeletal Infection Society (MSIS) criteria can miss some PJI cases, especially when caused by low-virulence microorganisms [29]. However, there is still a lack of data on the most appropriate criteria for defining PJI.

## Conclusion

Occasionally, elevated serum inflammatory markers can be the first indication of PJI and can initiate further diagnostic analysis. In this study, serum CRP and fibrinogen showed the best performances in comparison to the other analysed serum markers. However, their results should be interpreted with caution in clinical practice. They can often remain normal in chronic infections or can be elevated in patients with other inflammatory conditions. Although our evaluated biomarkers are easily accessible (even in patients with a dry joint aspiration) and are routinely evaluated pre-operatively without any additional cost, they showed insufficient performances in diagnosing PJI. Hence, it is clear, that they cannot be used alone to confirm PJI but can serve as suggestive criteria in diagnosing PJI. In clinical practice, the pre-operative workup should always be complemented by more specific tests such as synovial fluid analysis (leukocyte count, conventional culture).

## Data Availability

All data generated or analysed during this study are included in this published article.
